# Microscopic Colitis with Macroscopic Endoscopic Findings

**DOI:** 10.1155/2013/461485

**Published:** 2013-04-09

**Authors:** Atif Saleem, Parag A. Brahmbhatt, Sarah Khan, Mark Young, Gene D. LeSage

**Affiliations:** ^1^Division of Gastroenterology and Hepatology, Department of Internal Medicine, East Tennessee State University, P.O. Box 70622, Johnson City, TN 37614, USA; ^2^Department of Internal Medicine, East Tennessee State University, Johnson City, TN 37614, USA

## Abstract

Microscopic Colitis (MC) is characterized by chronic watery diarrhea, grossly normal appearing colonic mucosa during conventional white light endoscopy, and biopsy showing microscopic inflammation. We report a case of collagenous colitis with gross endoscopic findings.

## 1. Case Report 

**Figure 1:**
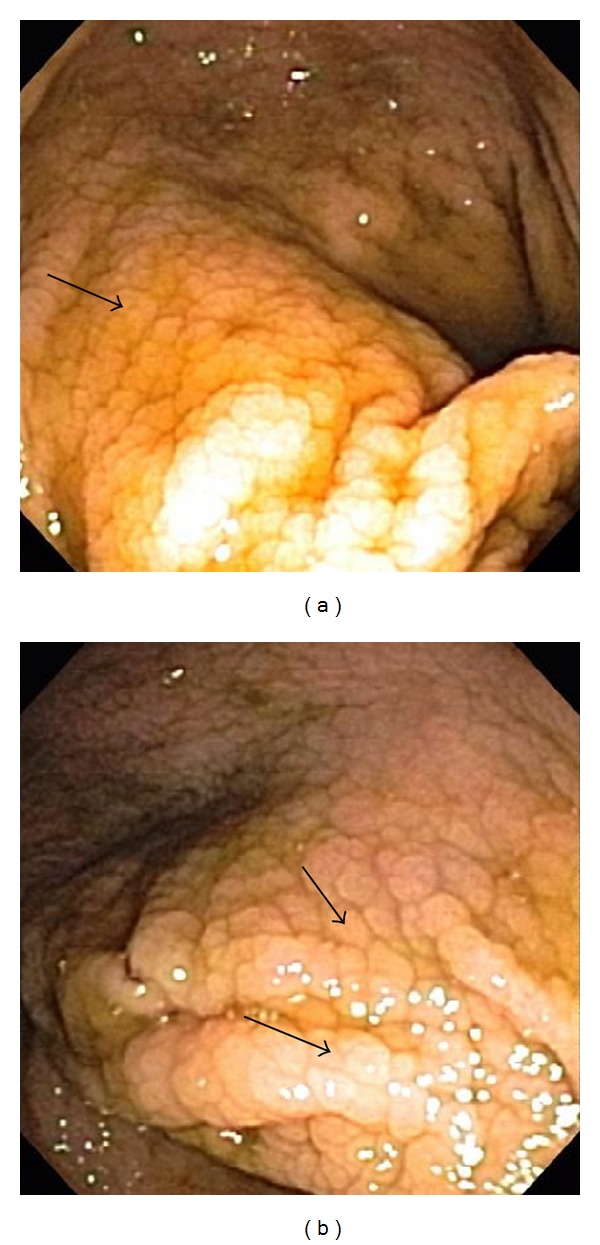
Colonoscopy images showing increased nodularity and loss of vascular markings.

A 71-year-old female with past medical history of coronary artery disease, carotid artery disease, hypertension, and diabetes mellitus type 2 was being evaluated for recurrent intermittent diarrhea. Patient denied any abdominal pain, weight loss, or any blood in stool. Patient was not on any medications and lab work was within normal limits. Colonoscopy was required to further evaluate patient's diarrhea. Colonoscopy revealed multiple scattered segments throughout the colon with increased nodularity and loss of vascular markings in the hepatic flexure, descending colon and cecum (Figures 1(a) and 1(b)). These findings were more pronounced in cecum. Colonoscopy findings were reported as nonspecific colitis involving scattered segments in the colon, consistent with a Crohn's disease. Multiple biopsies were taken from both affected and nonaffected areas of the colon for pathologic determination. 

**Figure 2:**
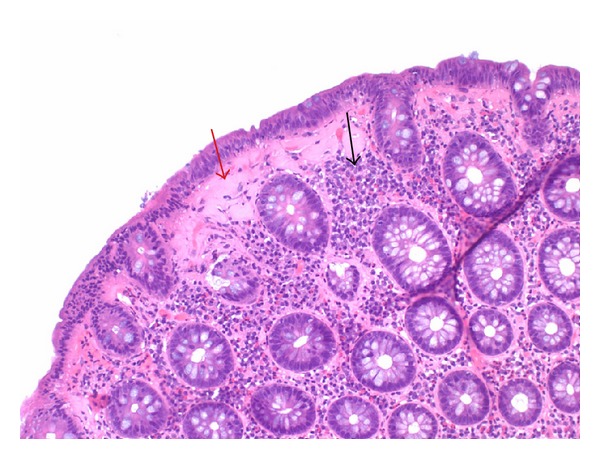
Show prominent thickening of the submucosal collagen table. The submucosal collagen is dense and paucicellular with several entrapped lymphocytes and capillaries (red arrow). The lamina propria display an increase in eosinophils, plasma cells, and lymphocytes (black arrow).

Microscopic examination showed lymphocytic colitis with marked thickening of the superficial collagen table consistent with collagenous colitis. The submucosal collagen was dense and paucicellular with several entrapped lymphocytes and capillaries (red arrow). The lamina propria displays an increase in eosinophils, plasma cells, and lymphocytes (black arrow) (Figure 2).

## 2. Discussion

Microscopic Colitis (MC) is characterized by chronic watery diarrhea, grossly normal appearing colonic mucosa during conventional white light endoscopy, and biopsy showing microscopic inflammation. It accounts for 4%–13% of patients evaluated for chronic diarrhea and divided into lymphocytic and collagenous colitis [1]. MC is usually considered disease of the elderly and patients with age of more than 65 years have five times higher risk for MC. Female predominance has been noted particularly for collagenous colitis [2]. Certain medications such as NSAIDs, PPIs, and SSRIs, as well as autoimmune disease such as thyroid disease, and celiac disease are associated with increased risk of MC [1, 2]. 

Although identical clinically, both types of colitis have distinct microscopic findings. Lymphocytic colitis is characterized by intraepithelial lymphocytosis (more than 20 lymphocytes for every 100 epithelial cells) while collagenous colitis is diagnosed by thickening of subepithelial collagen layer of more than 10 micrometer [2]. 

By definition colonic mucosa has a normal appearance in MC. Yet distinct endoscopic findings, particularly for collagenous colitis, have been described in the literature. These findings include alteration of mucosal vascular pattern, mucosal abnormalities such as red spots, increased mucosal nodularity, or textural alteration, and pseudomembranes [3]. 

Recent advance in endoscopy may enhance the ability to detect MC. A newly developed post processing light filters such as i-Scan (Pentax, Japan) helps to enhance the visualization of mucosal pattern and vascular architecture. Chromoendoscopy using indigocarmine dye sprays may show a mosaic pattern of mucosa in lymphocytic colitis and a nodular, grooved pattern in collagenous colitis [4]. These new techniques can increase the yield of endoscopic diagnosis of MC by targeted biopsy compared to random biopsies at conventional white light endoscopy [4]. 

## 3. Conclusion

Microscopic Colitis is traditionally known to have normal colonic mucosa on endoscopy. Recent advance in endoscopic techniques has shown that various mucosal abnormalities such as alteration of mucosal vascular pattern and increased mucosal nodularity are associated with MC. These newer techniques will allow us to get targeted biopsy, which will increase the yield of endoscopic diagnosis of MC.
